# Risk Factors for Fasciotomy After Revascularization for Acute Lower Limb Ischaemia

**DOI:** 10.3389/fsurg.2021.662744

**Published:** 2021-03-29

**Authors:** Emil Karonen, Axel Wrede, Stefan Acosta

**Affiliations:** ^1^Department of Clinical Sciences, Lund University, Malmö, Sweden; ^2^Vascular Center, Department of Cardiothoracic and Vascular Surgery, Skåne University Hospital, Malmö, Sweden

**Keywords:** acute limb ischaemia, acute compartment syndrome, fasciotomy, vascular surgery, gender

## Abstract

**Background:** Acute lower limb ischaemia (ALI) is a life and limb threatening vascular emergency. Acute compartment syndrome (ACS) may develop upon revascularization. The risk of fasciotomy was hypothesized to be decreased in women due to their lower calf muscle mass. The main aim was to evaluate risk factors for fasciotomy after revascularization for ALI.

**Methods:** This is a retrospective observational study of patients undergoing revascularization for ALI between 2001 and 2018. Factors associated with outcome at 1 year in univariable analysis (*p* < 0.1) were chosen for multi-variable analysis and expressed in Odds Ratios (OR) with 95% confidence intervals (CI).

**Results:** The median age for women (*n* = 394) was 75 years and men (*n* = 449) was 70 years (*p* < 0.001). The frequency of fasciotomy was 10.0% (84/843). The median in-hospital stay was 28 vs. 6 days for patients undergoing fasciotomy and not, respectively (*p* < 0.001). In adjusted analysis, renal insufficiency (OR 1.77, 95% CI 1.04–3.01), motor deficit (OR 4.40, 95% CI 2.45–7.92), popliteal artery aneurysm thromboembolism (OR 2.26, 95% CI 1.06–4.80), and open vascular surgery (OR 3.43, 95% CI 1.97–5.98) were associated with an increased risk of fasciotomy. Female patients (OR 0.49, 95% CI 0.28–0.84) and anemia (OR 0.52, 95% CI 0.28–0.84) had a lower risk. The major amputation/mortality rate at 1-year was 27.7%; fasciotomy (OR 1.94, 95% CI 1.11–3.40), anemia (OR 1.84, 95% CI 1.24–2.73) and female gender (OR 1.44, 95% CI 1.00–2.08) were independently associated with an increased risk.

**Conclusions:** Female patients had lower rates of fasciotomies, but subsequent higher risk of major amputation/mortality, which may be attributed to inferior results of revascularization. Lower muscle mass and underdiagnosis of ACS could also explain the lower frequency of fasciotomy for female patients. Further studies are needed to better understand gender differences in presentation of ALI, revascularization results and diagnosis of ACS.

## Introduction

Acute lower limb ischaemia (ALI) is caused by sudden onset of hypoperfusion of one or both lower extremities, most commonly a result of an arterial thrombosis or embolism. The symptoms must have appeared in the last 14 days to be considered ALI (1). If not treated urgently, ALI can cause muscle necrosis and irreversible nerve damage, limb gangrene and death. A complication after a revascularization procedure for ALI is acute compartment syndrome (ACS), where the ischaemic muscle cells are further damaged upon reperfusion (2). The muscles of the calf, in particular, can only expand to a certain degree within the tight fascia. In case of severe swelling of the calf within the tight fascia enveloping the muscles a therapeutic fasciotomy, by incisions of the four anatomic fascias, is necessary to avoid irreversible muscle necrosis. Compartment syndrome following reperfusion for ALI can be anticipated, whereby the vascular surgeon completes the primary revascularization procedure by a prophylactic fasciotomy before reperfusion injury to the muscles has occurred. If left untreated, ACS can lead to paralysis, limb gangrene, rhabdomyolysis and acute kidney injury (3). Even though fasciotomy can be life and limb saving it is associated with complications, above all wound complications and nerve damage (4). The hypothesis of the present study was that female gender due to their, in general, lower calf muscle mass (5) was a protective factor toward ACS and fasciotomy.

The main aim of this study was to evaluate risk factors for fasciotomy, and a secondary aim was to investigate risk factors associated with combined major amputation/mortality at 1 year, after reperfusion due to ALI. This gives an opportunity to relate risk factors for fasciotomy to risk factors for major amputation/mortality.

## Methods

This study was approved by the Swedish Ethical Review Authority (Dnr 2020/00764).

It is a retrospective observational study done on patients with ALI. The data was collected from patients undergoing revascularization for ALI from January 1st 2001 to December 31st 2018 at Vascular Center, Skåne University Hospital (SUS) Malmö, which is a tertiary referral center with a primary catchment population of ~1.25 million inhabitants in 2010. Computed tomography angiography and invasive angiography were disposable at all hours, and ultrasound lower extremity arteries and magnetic resonance imaging where available office hours.

### Revascularization

Patients underwent primarily open vascular or endovascular treatment. Open vascular treatment was usually balloon catheter thromboembolectomy, more seldom bypass procedures, or thrombendarterectomy. The majority of study patients underwent local continuous thrombolysis, seldom in combination with percutaneous mechanical thromboembolectomy. Local intra-arterial thrombolysis was initiated by arterial puncture in the common femoral artery, usually in the non-diseased leg, advancing a long thrombolysis catheter, equipped with multiple side holes, over the aortic bifurcation to the diseased leg, positioned in the occlusion to be able to deposit high concentration of alteplase (Actilyse; Boehringer Ingelheim, Stockholm, Sweden). Initially 1–2 mg/h was administered during the first 4 h, followed by 0.5–1.0 mg/h. In parallel, a bolus dose of heparin (Heparin Leo, LEO Pharma, Malmö, Sweden) followed by continuous local administration through a side hole of the introducer. Since 2012, patients receiving local thrombolysis were sometimes treated at the ward instead of intensive care unit, and concomitant heparin infusion was abandoned.

### Fasciotomy Wound Treatment

The fasciotomy wounds were dressed using negative pressure wound therapy dressings (NPWT) or compresses and gauze dressings. NPWT was started at the time of fasciotomy or the day after, after wound revision, or when it was deemed necessary. A black poly urethane or white polyvinyl alcohol sponge (KCI Medical, San Antonio, Texas, USA) was applied with a topical negative pressure of 125 mm Hg. Changes of NPWT dressings were usually performed three times per week. The first re-dressings were sometimes performed at the operation theathre. Sometimes NPWT was applied using white polyvinyl alcohol sponge on top of a fresh split skin graft transplant.

### Variable Definition

Severity of ALI was graded according to the Rutherford Criteria (6). The decision to perform fasciotomy was down to the treating surgeon and not based on any specific criteria. A fasciotomy was considered as therapeutic if there were noted signs of ACS when it was performed, otherwise it was considered as prophylactic. Major amputation was defined as any amputation of the leg above the ankle. Major bleeding was defined as a bleeding where there was a need for blood transfusion with at least 2 units of erythrocytes, had thrombolysis canceled because of bleeding or had to undergo reoperation due to bleeding. The in-hospital stay was the total amount of days the patients stayed at the hospital in association with the event of ALI. Renal insufficiency was defined as a serum creatinine level of higher than 105 μg/L for male and 90 μg/L for female patients. Anemia was defined by a hemoglobin level of lower than 134 mg/L for male and 117 mg/L for female patients. Ischemic heart disease was defined as a history of myocardial infarction or coronary artery bypass grafting or percutaneous coronary intervention. Cerebrovascular events were defined by a patient previously having a stroke or transient ischemic attack.

### Statistical Analysis

For data analysis SPSS Statistics version 26 (IBM, Armonk, NY, USA) was used. Variables were expressed in proportions (percentages) or median and inter-quartile range (IQR). For group comparisons with nominal and continuous data, Pearson's chi square and Mann Whitney *U*-test were performed, respectively. Risk factors for fasciotomy and factors associated with combined major amputation/mortality at 1 year in univariable analysis (*p* < 0.1) were chosen for multi-variable logistic regression analysis and expressed in Odds Ratios (OR) with 95% confidence intervals (CI). *P*-values < 0.05 were considered significant.

## Results

### Patient Characteristics at Admission

Among 843 revascularization procedures for ALI, 84 (10.0%) underwent fasciotomy. Median age was 72 years (IQR 65–80) for the whole cohort, 75 years (IQR 68–82) in women (*n* = 394) compared to 70 years (IQR 61–77) in men (*n* = 449) (*p* < 0.001). Male patients underwent more frequently fasciotomy (12.5%) in comparison to female patients (7.1%) (*p* = 0.009). Patients that received fasciotomies presented more often with renal insufficiency at admission (46.3%) than non-fasciotomized patients (30.5%) (*p* = 0.004) (Table 1).

**Table 1:** Patient characteristics at admission in patients undergoing fasciotomy or not after reperfusion for ALI.

	**All** **(*n* = 843)**	**Fasciotomy** **(*n* = 84)**	**No Fasciotomy** **(*n* = 759)**	***p*-value**
Admittance 2001–2009 (%)	379 (45.0)	24 (6.3)	355 (93.7)	
Admittance 2010–2018 (%)	464 (55.0)	60 (12.9)	404 (87.1)	0.001
Age, median (IQR)	72 (65–80)	71.5 (64.5–79)	72 (65–80)	0.59
Female (%)	394 (46.7)	28 (7.1)	366 (92.9)	
Male (%)	449 (53.3)	56 (12.5)	393 (87.5)	0.009
**Comorbidities (%)**				
Hypertension	656 (77.8)	63 (75)	593 (78.1)	0.51
Diabetes mellitus	294 (22.9)	19 (22.6)	174 (22.9)	0.95
Atrial fibrillation (*n* = 842)	234 (27.8)	27 (32.1)	207 (27.3)	0.35
Ischemic heart disease	267 (31.7)	30 (35.7)	237 (31.2)	0.40
Cerebrovascular disease	147 (17.4)	16 (19.0)	131 (17.3)	0.68
Claudication (*n* = 836)	429 (51.3)	36 (43.4)	393 (52.2)	0.13
**Medical therapy at admission (%)**				
ASA (*n* = 842)	446 (53.0)	40 (47.6)	406 (53.6)	0.30
Clopidogrel (*n* = 842)	81 (9.6)	6 (7.1)	75 (9.9)	0.42
Warfarin, full dose LMWH or DOAC	118 (14.0)	13 (15.5)	105 (13.8)	0.68
**Laboratory data at admission**				
Renal insufficiency (%) (*n* = 832)	267 (32.1)	38 (46.3)	229 (30.5)	0.004
Anemia (%) (*n* = 827)	226 (27.3)	15 (18.8)	211 (28.2)	0.070
CRP level, median (IQR) (*n* = 757)	9.0 (4.0–31.0)	9.0 (3.8–31.0) (*n* = 75)	9.0 (4.0–31.0) (*n* = 682)	0.68

ALI, acute lower limb ischemia; IQR, interquartile range; ASA, acetyl salicylic acid; LMVH, low molecular weight heparin; DOAC, direct oral anticoagulants; CRP, C-reactive protein.

### Popliteal Artery Aneurysm

There were 59 male patients (13.1%) with popliteal artery aneurysm (PAA) among 449 men, compared to five female patients (1.3%) out of 394 women (*p* < 0.001). There were 6 (3.1%) patients with PAA among 193 patients with diabetes mellitus, compared to 58 (8.9%) among 650 patients without diabetes (*p* = 0.007). ALI secondary to PAA was caused by thrombotic occlusion within the aneurysm in 71.9% (*n* = 46) and embolism to the calf arteries from the non-occlusive thrombosis within the aneurysm in 28.1% (*n* = 18).

### Diagnostic Assessment

Patients that received fasciotomies had a shorter symptom duration from onset of symptoms to operation (*p* < 0.001), lower ABI (*p* < 0.001), more often Rutherford IIB ALI (motor deficit) (*p* < 0.001), bilateral ALI (*p* < 0.001) and more often popliteal artery aneurysm (PAA) (*p* < 0.001) than the non-fasciotomized patients (Table 2).

**Table 2:** Diagnostic assessment at admission in patients undergoing fasciotomy or not after reperfusion for ALI.

	**All** **(*n* = 843)**	**Fasciotomy** **(*n* = 84)**	**No fasciotomy** **(*n* = 759)**	***p*-value**
**Vascular status of affected limb(s)**				
Foot ulcers (%) (*n* = 840)	101 (12.0)	7 (8.3)	94 (11.2)	0.27
Symptom duration (hours), median (IQR) (*n* = 816)	48 (16–120)	24 (9–72) (*n* = 83)	48.0 (19–120) (*n* = 733)	<0.001
Ankle-brachial index, median, IQR (*n* = 670)	0.0 (0.0–0.0)	0.0 (0.0–0.0) (*n* = 55)	0.0 (0.0–0.11) (*n* = 615)	<0.001
Rutherford class IIb (motor deficit) (*n* = 842)	307 (36.5)	63 (75.0)	244 (32.2)	<0.001
**Level and localization of occlusion(s) (%)**				
Bilateral arterial occlusions	54 (6.4)	13 (24.1)	41 (5.4)	<0.001
Supra-inguinal occlusion	276 (32.7)	29 (34.5)	247 (32.5)	
Infra-inguinal occlusion	567 (67.3)	55 (65.5)	512 (67.5)	0.71
**Type of occlusion (%)**				
Native artery occlusion	500 (59.3)	57 (67.9)	443 (58.4)	0.093
Bypass graft occlusion	153 (18.1)	12 (7.8)	141 (18.6)	0.18
Venous bypass graft occlusion	37 (4.4)	3 (3.6)	34 (4.5)	0.70
Synthetic bypass graft occlusion	118 (14.0)	9 (10.7)	75 (14.4)	0.36
Stent/stentgraft occlusion	192 (22.8)	14 (16.7)	178 (23.5)	0.16
**Etiology of occlusion (%)**				
Thrombus	208 (24.7)	19 (9.1)	189 (24.9)	0.64
Embolus	234 (27.8)	211 (27.8)	23 (27.4)	0.94
PAA occlusion	64 (7.6)	15 (17.9)	49 (6.5)	<0.001
**Any imaging pre thrombolysis (%)**	570 (67.6)	59 (70.2)	511 (67.3)	0.59

*ALI, acute lower limb ischemia; IQR, interquartile range; PAA, popliteal arterial aneurysm*.

### Revascularization and Fasciotomy

Patients undergoing fasciotomies had more often primary open vascular surgery compared to endovascular therapy (*p* < 0.001), more major bleeding (*p* < 0.001), and a longer hospital stay (*p* < 0.001) than the non-fasciotomized (Table 3). Patients undergoing prophylactic compared to therapeutic fasciotomy had more often Rutherford IIb (*p* < 0.001), renal insufficiency (*p* = 0.031) and were more often operated with open vascular surgery (*p* < 0.001) (Table 4). NPWT was used at some time point during the fasciotomy treatment in 51.4% and split skin graft was used in 40.8%.

**Table 3:** Treatment, complications, and short-term outcome in patients undergoing fasciotomy or not after reperfusion for ALI.

	**All** **(*n* = 843)**	**Fasciotomy** **(*n* = 84)**	**No fasciotomy** **(*n* = 759)**	***p*-value**
**Treatment**				
In-hospital stay (days), median (IQR) (*n* = 676)	7 (5–13)	28 (13–51) (*n* = 76)	6 (4–11) (*n* = 600)	<0.001
Primary endovascular treatment (including thrombolysis) (%)	595 (70.6)	30 (35.7)	565 (74.4)	<0.001
Primary open vascular surgery (%)	227 (26.9)	52 (61.9)	175 (23.1)	<0.001
Adjunctive (open or endovascular) surgery (%) (*n* = 842)	609 (72.3)	60 (71.4)	549 (72.4)	0.85
**Complications during in-hospitalization (%)**				
Major bleeding (*n* = 840)	189 (22.5)	40 (47.6)	149 (19.7)	<0.001
**Outcome at 30-day follow-up (%)**				
Major amputation	72 (8.6)	10 (12.2)	62 (8.2)	0.22
Mortality (*n* = 840)	41 (4.9)	6 (7.2)	35 (4.6)	0.30
Amputation-free survival (*n* = 838)	732 (87.4)	68 (82.9)	664 (87.8)	0.20
**Outcome at 1-year follow-up (%)**				
Major amputation (*n* = 839)	83 (9.9)	18 (14.4)	65 (9.1)	0.067
Mortality (*n* = 841)	83 (9.9)	24 (17.4)	59 (8.4)	0.001
Amputation-free survival (*n* = 841)	608 (72.3)	45 (54.2)	563 (74.3)	<0.001

ALI, acute lower limb ischemia; IQR, interquartile range.

**Table 4:** Uni-variable analysis of factors associated with prophylactic or therapeutic fasciotomy after revascularization for ALI.

**Variable**	**Timing of fasciotomy**		
	**Prophylactic (%)**	**Therapeutic (%)**	***p*-value**
Percentage of fasciotomies	52.6	47.4	-
Primary open vascular surgery	75.0	25.0	<0.001
Endovascular surgery	4.2	95.8	<0.001
Female	62.5	37.5	0.24
Anemia	69.2	30.8	0.21
Renal insufficiency	66.7	33.3	0.031
Rutherford IIb	63.8	36.2	<0.001
Bilateral arterial occlusions	76.9	23.1	0.054
PAA	46.2	53.8	0.61
Major amputation/mortality at 1 year	68.6	31.4	0.013

*ALI, acute lower limb ischemia; PAA, popliteal artery aneurysm*.

### Major Amputation and Mortality

There were no differences in 30-day major amputation/mortality rate between those undergoing fasciotomy or not. The 1-year mortality was 17.4% in the fasciotomy and 8.4% in the non-fasciotomy group (*p* = 0.001). There was a trend that major amputation at 1 year was higher in the fasciotomy group (*p* = 0.067). The amputation-free survival at 1 year was higher in the non-fasciotomy group (*p* < 0.001) (Table 3).

### Factors Associated With Fasciotomy

Prophylactic and therapeutic fasciotomies were performed in 52.6 and 47.4% of the patients, respectively. Four compartment-fasciotomy in the lower leg were performed in 92.1% of patients.

After multi-variable logistic regression, admittance 2010–2018 (OR 1.92, 95% CI 1.09–3.39, *p* = 0.024), renal insufficiency (OR 1.77, 95% CI 1.04–3.01, *p* = 0.034), Rutherford IIb ALI (OR 4.40, 95% CI 2.45–7.92, *p* < 0.001), PAA (OR 2.26, 95% CI 1.06–4.80, *p* = 0.035), and open vascular surgery (OR 3.43, 95% CI 1.97–5.98) were associated with an increased risk of fasciotomy. Female gender (OR 0.49, 95% CI 0.28–0.84, *p* = 0.010) and anemia (OR 0.52, 95% CI 0.28–0.84, *p* = 0.046) were associated with a lower risk of fasciotomy (Table 5).

**Table 5:** Factors associated with fasciotomy after revascularization for ALI.

**Variable**	**Multi-variable logistic regression**	
	**OR (95% CI)**	***p*-value**
Admittance 2001–2009		
Admittance 2010–2018	1.92 (1.09–3.39)	0.024
Primary open vs. endovascular surgery	3.43 (1.97–5.98)	<0.001
Female	0.43 (0.24–0.76)	0.004
Anemia	0.43 (0.22–0.83)	0.012
Renal insufficiency	1.77 (1.04–3.01)	0.034
Rutherford IIb	4.40 (2.45–7.92)	<0.001
Bilateral arterial occlusions	1.38 (0.62–3.11)	0.43
PAA	2.26 (1.06–4.80)	0.035

ALI, acute lower limb ischemia; PAA, popliteal artery aneurysm; OR, Odds Ratio; CI, Confidence Interval.

### Factors Associated With Combined Major Amputation and Mortality at 1-Year Follow Up

Patients with cerebrovascular disease (OR 1.86, 95% CI 1.23–2.82, *p* = 0.003), anemia (OR 1.84 95% CI 1.24–2.73; *p* = 0.002), foot ulcers (OR 2.47, 95% CI 1.46–4.18; *p* = 0.001), Rutherford IIb (OR 2.43, 95% CI 1.66–3.56; *p* < 0.001), infrainguinal occlusion (OR 1.62, 95% CI 1.07–2.46; *p* = 0.024), female gender (OR 1.44, 95% CI 1.00–2.08; *p* = 0.049), major bleeding (OR 1.94 95% CI 1.30–2.90; *p* = 0.001) and fasciotomy (OR 1.94, 95% CI 1.11–3.40; *p* = 0.021) were associated with major amputation/mortality at 1 year follow up (Table 6).

**Table 6:** Factors associated with combined major amputation/mortality at 1-year follow up in patients undergoing open or endovascular revascularization for ALI.

**Variable**	**Number of patients**	**Major amputation/mortality at 1 year (%)**	***p*-value**	**Multi-variable regression analysis**	
				**OR (95% CI)**	***p*-value**
All	843	231 (27.5)			
Admittance 2001–2009	379 (45.1)	117 (30.9)			
Admittance 2010–2018	461 (54.9)	114 (24.7)	0.047	0.77 (0.54–1.10)	0.15
Age ≥75 years	361 (42.8)	124 (34.3)	<0.001	1.27 (0.86–1.86)	0.23
Female	392 (46.7)	126 (32.1)	0.005	1.44 (1.00–2.08)	0.049
Hypertension	654 (77.9)	180 (27.5)	0.99		
Diabetes mellitus	192 (22.9)	61 (31.8)	0.13		
Ischemic heart disease	265 (31.5)	86 (32.5)	0.029	1.14 (0.78–1.67)	0.50
Cerebrovascular disease	146 (17.4)	56 (38.4)	0.001	1.86 (1.23–2.82)	0.003
Atrial fibrillation	233 (27.8)	86 (36.9)	<0.001	1.38 (0.89–2.15)	0.15
Claudication (*n* = 833)	428 (51.4)	112 (26.2)	0.42		
**Medical therapy**					
ASA	445 (53.0)	109 (24.5)	0.036	0.86 (0.61–1.26)	0.47
Clopidogrel	81 (9.7)	25 (30.9)	0.48		
Anticoagulation	118 (14.0)	42 (35.6)	0.034		
**Status at admission**					
Renal insufficiency	266 (32.1)	89 (33.5)	0.006	1.12 (0.78–1.62)	0.54
Anemia	226 (27.4)	86 (38.1)	<0.001	1.84 (1.24–2.73)	0.002
Foot/leg ulcers	100 (11.9)	39 (39.0)	0.005	2.47 (1.46–4.18)	0.001
Rutherford IIb	304 (36.2)	122 (40.1)	<0.001	2.43 (1.66–3.56)	<0.001
**Occlusion characteristics**					
Supra-inguinal occlusion	276 (32.9)	60 (21.7)			
Infra-inguinal occlusion	564 (67.1)	171 (30.3)	0.009	1.62 (1.07–2.46)	0.024
Native artery occlusion	501 (59.6)	156 (31.1)	0.003	0.98 (0.58–1.66)	0.94
Bypass graft occlusion	152 (18.1)	41 (27.0)	0.003		
Stent/stentgraft occlusion	192 (22.9)	34 (17.7)	0.001	0.74 (0.41–1.33)	0.31
Thrombus	207 (24.6)	60 (29.0)	0.58		
Embolus	233 (27.7)	77 (33.0)	0.026	0.88 (0.56–1.40)	0.60
PAA occlusion	64 (7.6)	16 (25.0)	0.641		
**Treatment/complication**					
Endovascular surgery	614 (73.1)	147 (23.9)			
Open vascular surgery	226 (26.9)	84 (37.2)	<0.001	1.06 (0.69–1.62)	0.79
Adjunctive any surgery	608 (72.4)	148 (24.3)	0.001		
Major bleeding	188 (22.5)	82 (43.6)	<0.001	1.94 (1.30–2.90)	0.001
Fasciotomy	83 (9.9)	38 (45.8)	<0.001	1.94 (1.11–3.40)	0.021

*ALI, acute lower limb ischemia; OR, Odds Ratio; CI, confidence interval; ASA, acetyl salicylic acid; LMWH, low molecular weight heparin; DOAC, direct oral anticoagulants; PAA, popliteal artery aneurysm*.

Patients undergoing prophylactic compared to therapeutic fasciotomy had higher rate of major amputation/mortality at 1 year (*p* = 0.013) (Table 4). When entering open vs. endovascular surgery, Rutherford IIb, and prophylactic vs. therapeutic fasciotomy in a multi-variable regression analysis, prophylactic fasciotomy (OR 5.9, 95% CI 1.4–25.8; *p* = 0.017) remained as an independent factor associated with major amputation/mortality at 1 year.

## Discussion

The present study identified several risk factors associated with increased and decreased risk of fasciotomy that may be useful in the management of patients undergoing revascularization for ALI. It was expected to find out that Rutherford IIb (motor deficit) ALI was associated with fasciotomy, which is related to the reperfusion injury imposed to the severely ischaemic skeletal muscles in the lower leg. The increased risk of fasciotomy in the latter half of the study period is unclear, but might be associated with a higher awareness of ACS by surgeons and/or undertaking more revascularization procedures in patients with more advanced ALI.

Female patients underwent fasciotomies more rarely than male patients, which might be associated with their higher age and, in general, lower muscle mass in the calves and perhaps also more relaxed fascia. Hence, ACS might develop more insidious and be more reversible. A study based on a small case series has suggested that prophylactic fasciotomies could be avoided in elderly female patients, even in longer time periods of ALI, due to their in general lower muscle mass (7). Another plausible reason for the lower risk of fasciotomy in females may be that clinical symptoms and signs of ACS are less clear, leading to underdiagnosis of ACS and fewer fasciotomies. A third reason may be related to an inferior result of the revascularization procedure compared to men, resulting in less need of fasciotomies.

The finding that anemia was associated with decreased risk of fasciotomy may be explained by that anemia could be interpreted as a proxy for poor general health (8, 9) and aging (10), as such, also lower muscle mass.

The increased risk for fasciotomy among patients with ALI due to PAA may be explained by a severe ischaemic state in a typically male patient with greater muscle mass undergoing clearance of embolic occlusions of often several calf arteries resulting in major reperfusion after revascularization. Secondly, patients with PAA often have mild atherosclerotic occlusive lesions and a rather good ankle-brachial index prior to falling ill (11), and a low frequency of diabetes mellitus (12), as shown in the present study, further, facilitating larger reperfusion volumes. Third, acute open repair of a PAA followed by fasciotomy, partly through the same incision, is easily performed before closure of the operation, slightly lengthening the procedure.

Identification of renal insufficiency at admission was probably most related to acute kidney injury due to dehydration and not to chronic renal failure, but this factor was nevertheless found to be associated with increased risk for fasciotomy. Presence of renal insufficiency may be a sign of vulnerability toward accumulation of fluids, and in combination with intravenous administration of high amounts of crystalloid fluids in the perioperative period, may facilitate the development of ACS and need for fasciotomy. A previous study suggests that a higher positive fluid balance was associated with development of compartment syndrome in patients undergoing treatment for ALI (2). A canine study demonstrated that fluid resuscitation after hypovolemic shock increased intra-compartmental pressure even without local trauma (13), suggesting that administration of high amounts of intravenous fluids after reperfusion of an ischemic limb could increase the intra-compartmental pressure compared to controls. This notion makes physiologically sense as well with a low amount of administered intravenous fluids remaining in the circulatory system. With muscle necrosis, intracellular proteins, and electrolytes are exudated in the intra-compartmental area, increasing the osmotic pressure leading to fluid migration. Inflammation in the area leads to increased vascular permeability. This cocktail of factors could worsen the already poor fluid distribution from the administered fluids and increase the risk of developing ACS.

Female gender was associated with a higher risk of combined major amputation and mortality at 1 year follow up. The higher rate of major amputation and mortality in female patients undergoing open (14) or endovascular (15) surgery for ALI has been reported previously. The differences between gender could be several, such as, estimation of symptoms and severity of disease, hormonal, anatomical, iatrogenic damage, delayed diagnosis, inferior, and delayed treatment. The smaller arteries in females compared to males may result in that the arteries may be more susceptible to iatrogenic injuries (16, 17). The worse outcome for female patients with ALI may be a result of inferior results of the revascularization procedure. Female patients were older than male patients, and age may have contributed to adverse outcome, even if age not was associated with higher combined major amputation and mortality at 1 year in the multi-variable regression analysis. It could also be speculated that female patients who develop ALI may possess a higher disease burden than male patients and thus be more fragile. Taken together, strategies for management of female patients undergoing vascular and endovascular surgery for ALI needs to be further defined to improve outcome.

The link between cerebrovascular disease, anemia, and foot ulcers at admission and increased risk of major amputation/mortality at 1 year could be explained by poor general health and increased fragility at baseline. The association between Rutherford IIb and combined major amputation/mortality could be explained by the severity of ALI at admission. The association between infra-inguinal occlusion and combined major amputation and mortality might be because of surgical difficulty in revascularization. The association between fasciotomy and major amputation/mortality could be explained with focus on three aspects. Patients with severe symptoms and signs of ALI often receive prophylactic fasciotomies. The remaining patients that receive fasciotomies have developed ACS. Both these groups should have an increased risk of major amputation and mortality. In addition, fasciotomies are associated with prolonged in-hospital stays, wound complications, difficulties in mobilization resulting in hospital-acquired complications such as pneumonia and venous thromboembolism (18). Prophylactic fasciotomy was in an analysis adjusted for Rutherford IIb, open vascular as opposed endovascular surgery, independently found to be associated with increased risk of major amputation/mortality at 1 year in the present study. This finding is in line with a recent study (4) and suggests that vascular surgeons perceived these patients as high risk for dismal prognosis.

The main limitation of this study was the retrospective observational design. Prospective studies adhering to a predefined protocol are challenging to conduct but necessary in order to evaluate the association between specific plasma biomarkers and severity of ALI, risk of ACS after reperfusion and fasciotomy. The plasma biomarkers matrix metalloproteinases and neutrophil gelatinase-associated lipocalin were found to be elevated at admission and maintained elevated after reperfusion and fasciotomy, compared to a control group not undergoing fasciotomy (19), but these markers independent diagnostic value for severity of ALI and ACS, and prognosis, apart from clinical variables such as presence of Rutherford IIb, need further evaluation.

The major strength of the present study was the large size of cohort with ALI undergoing revascularization and the rather high rate of patients undergoing fasciotomy. This gives an opportunity to adjust for confounding factors in the statistical analysis. In comparison to other studies including both acute and electively treated vascular surgical patients (4, 20), the present study included exclusively patients undergoing revascularization for ALI.

## Conclusions

Female patients had lower rates of fasciotomies, but subsequent higher risk of major amputation/mortality, which may be attributed to inferior results of revascularization. Lower muscle mass and underdiagnosis of ACS could also explain the lower fasciotomy frequency for female patients. Further, studies are needed to better understand gender differences in presentation of ALI, revascularization results and diagnosis of ACS.

## Data Availability Statement

The raw data supporting the conclusions of this article will be made available by the authors, without undue reservation.

## Ethics Statement

The studies involving human participants were reviewed and approved by Swedish Ethical Review Authority (Dnr 2020/00764). Written informed consent for participation was not required for this study in accordance with the national legislation and the institutional requirements.

## Author Contributions

EK was involved in the design of the study, data gathering, data analysis, and writing of the manuscript. AW was involved in data gathering and critical review of the manuscript. SA was involved in design of the study, data gathering, data analysis, and critical review of the manuscript. All authors contributed to the article and approved the submitted version.

## Conflict of Interest

The authors declare that the research was conducted in the absence of any commercial or financial relationships that could be construed as a potential conflict of interest.
